# Thirty-Seventh Annual Meeting of the Mississippi Valley Dental Society

**Published:** 1881-04

**Authors:** 


### THIRTY-SEVENTH ANNUAL MEETING OF THE MISSISSIPPI VALLEY DENTAL SOCIETY.

### DISCUSSION OF THE SUBJECTS.

*Reported for the Register.*

The Mississippi Valley Dental Society held its thirty-seventh annual meeting at the Ohio College of Dental Surgery, Cincinnati, commencing Wednesday morning, March 2d, 1881, and proceeded to discuss the subjects presented by the Executive Committee.

First subject. Alveolar abscess, and treatment.

The discussion was opened by Dr. J. Taft, of Cincinnati. He laid down as a general principle that abscess in whatever tissue occurring, is the result of inflammation.

For the proper comprehension of the subject before us, it is necessary to understand the nature of the tissue in which the abscess exists, as well as the peculiarity of the patient, in as much as it varies in different subjects and tissues. It is to be observed that the irritation is slight in some cases, while in others the causes leading to it are violent, producing a corresponding degree of irritation. The first thing necessary to be ascertained is the chararcter of the irritant. The next step is to ascertain the amount of irritation produced by the irritant, and the determination of blood to the part. Any violent mental agitation or activity will cause an increased flow of blood to the brain. An increased flow in the irritated part may precede irritation. The circulation may be increased or diminished during an inflammatory attack, the red blood corpuscles being more or less detained in their passage by adhesion to the walls of the capillaries.

Exudation of the watery portion of the blood now takes place into the contiguous tissues, swelling resulting from that cause as well as from the congestion of the blood vessel. Stoppage of the circulation does not occur until after exudation, and it is only in an engorged condition of the parts that we have true stasis.

Up to this or any previous stage of the inflammation resolution may occur.

The step following stasis is suppuration, where this occurs there is a greater or less breaking down of the tissues. Suppuration may be confined to a small point, but in a more susceptible patient it will occupy a larger territory.

All tissues, including bone, suffer from the suppuration, the bone itself breaking down under it.

There is a theory that the cells found in pus are the white corpuscles of the blood which have passed through the walls of the blood vessels during exudation. In a copious discharge from a large abscess, it seems incredible that the blood could furnish so many of these corpuscles, when the fact is that they are comparatively few in number. It is probable that the pus cells also come from the cell formation of the neighboring tissues.

An alveolar abscess may confine itself to the socket of the tooth, or may break through the process and diffuse itself to a large extent through the contiguous soft parts. If a large extent of tissue is involved, one course of treatment is indicated; if small, another, though the pain may be excessive. An inflammation may terminate in resolution, suppuration, or become chronic. In the chronic form the tissues do not succumb, it being merely a subsidence from the active state. The hardness of the parts in the chronic state is caused by the coagulation of exuded lymph. This condition continues, sometimes for a long period, but eventually breaks down. The absorbents being in a debilitated condition require some lapse of time to carry away the products of exudation. To stimulate or hasten that action, iodine has been applied for some time, before an appreciable effect is obtained

Dr. Frank Hunter asked if pus formed, must there of necessity be an exit for it.

Dr. Taft replied in the negative.

The inflammation in the surrounding tissue may subside, and the pus be absorbed without any disturbance, or it may putrify, become absorbed into th blood causing septicemia. He did not believe in the pyogenic membrane as commonly understood, for it is merely the exudation in a coagulated condition, and an abortive attempt at reproduction.

The watery constitutents of the pus are absorbed, leaving a cheese-like pus which in its turn is thinned out and absorbed.

Dr. W. D. Kempton, of Cincinnati, said that an inflammation is a condition of hyper-nutrition. The lymph that exudes is susceptible of organization, the new tissue stimulating that tissue in which the exudation takes place. The centre of the inflammation is at the point where pus forms, an attempt at organization being made at the periphery. Where these processes meet is the place where, what is called, the pyogenic membrane, is formed.

Dr. J. Taft did not think that there was over nourishment in the parts, for in hypertrophied cementum it is difficult to distinguish the original from the newly formed material.

Dr. Kempton said that in the pleura of the lungs a seperate tissue may be formed between the layers, though this tissue is completely or perfectly formed.

Dr. James Taylor, of Cincinnati, desired to have some things about the sac-membrane explained.

Dr. J. Taft said in reply, that the periosteum had nothing to do with the formation of the sac, so-called. The periostium is usually destroyed during an abscess, sometimes it remains intact, it may be partially destroyed, or it may disappear altogether.

Dr. James Taylor said that the coagulation of the exudation forms a membrane, the pus forcing it from around the root. To treat an abveolar abscess we must understand systemic conditions. Sometimes a discharge of the pus is all that is necessary; in others a debilitated system needs constitutional treatment. He had known cases tainted with syphilis to resist all efforts at cure, and then he asked the question how such cases were to be handled?

Dr. J. Taft said that it was necessary to know whether the recuperative force was good or not. If the cause producing the abscess can be recognized, its removal will in many cases result in a cure. The tooth itself is sometimes the irritant, although there is no putrescent matter in it. Sometimes malarial poisoning may be an incidental cause preventing restoration. In a scrofulous person the tissues possess but little tone. As to syphylitic cases, if the dentist is not competent to treat them, the physician ought to be consulted.

Dr. Kempton said that after suppuration the exudate is organized by granulation. Lines appear in the coagulated exudation, and are the future blood vessels, nerves, &c.

Dr. J. S. Cassidy said that the treatment of abscess is by means of escharotics, all differing in power; and asked what would be the treatment proper for dead teeth which had already undergone a course of treatment.

Dr. Kempton replied that the abscess could be treated by making an opening through the gum and process.

Dr. Cassidy said, that if the abscess was obstinate and treatment of no avail, he would extract.

Dr. W. F. Morrill thought very favorably of boracic acid as a disinfectant. It is efficacious if the case is not of an obstinate character.

Dr. J. W. Jay, of Richmond, Ind., said that his method was after cleansing the tooth to introduce a bit of cotton impregnated with carbolic acid into the root, and pushing it up with soft rubber, in order to force the disinfectant through the fistulous opening. A good injection may be made by mixing carbolic acid, glycerine and pure water.

Dr. G. W. Keely said that in a syphilitic case if he could not cure the abscess he would extract the tooth. Scrofulous cases are very stubborn, the tooth producing trouble even after it had been treated and filled. With an ordinary abscess half the work is done when an opening is made through the end of the root, to establish communication with the fistula. He objected to over-treatment. If there was opening at the end of the root he would close it with gutta percha; if small, a solution of it would suffice. It is wrong to inject carbolic acid, if there is no fistula, for there the fluid goes through without producing irritation. He would like to know if the abscess itself is not an effort of nature, to prevent the absortion of putrifying matter in the canal. He cited a case of twenty years duration, in which the pus was discharged through the nose. In cutting through the palatal surface he found a large opening at the end of the root. The tooth was cleansed with injections of water and stopped with gutta percha, the case resulting favorably.

Dr. J. Taft said, that an abscess sometimes heals without any treatment. Frequently the causes producing the abscess cannot be reached, the trouble necessarily continuing. A deposit on the root sometimes is the cause, and therefore difficult or impossible to reach. Treatment with escharotics is not always indicated. And he objected to over-treatment. If the periosteum is involved it may serve to keep up the suppuration. Carbolic acid acts as a stimulant and also coagulates albumen. Gentle stimulation is the proper method after the debris is removed. If there is a fistulous opening, stop the opening at the end of the root and treat through the fistula.

Dr. H. A. Smith said that a chronic abscess showed the inability of the system to restore the parts to health. In such cases cut away the diseased tissue or destroy it with caustics, so as to bring about active inflammatory action. It is almost if not quite impossible to cure an abscess for a consumptive or anæmic patient.

Dr. J. Taft said that in consumption the lungs are unable to ærate the blood sufficiently; the tissues in consequence lose their vigor and cannot readily recuperate.

Dr. W. S. How cited a case of obstinate abscess in a consumptive. The discharge of pus was large, the boney walls being honey-combed. He treated the case with nitrate of silver and creosote.

Dr. Jay observed that extraction is always a sure cure.

Second Subject.—Treatment of superficial caries of the teeth.

Discussion of this subject was opened by Dr. Jay asking what was meant by the term "superficial caries?"

Dr. G. W. Keely replied that it was a slight decay, mostly seen on the proximate surfaces of the teeth. Wedge them apart and cut away the decay with a file and where possible the disk may be used, smoothing or finishing the surfaces with corrundum tape. If the teeth should remain sensitive after the operation, cauterize with nitrate of silver. He would enjoin upon the patient the necessity of absolute cleanliness as a means of success for this method of treatment.

Dr. J. Taft said that the majority of the profession leave the cleaning of the mouth to the last, when in reality it should be the first step to put a mouth in order, to thoroughly remove all deposits found upon the teeth. Then look for superficial decay, for failure in this respect is due to not finding the decay in the first place. For separating the teeth use the Jarvis Separator, then the disk, corundum tape, emery paper, &c., which will accomplish the purpose.

Third Subject.—To what extent does vitality resist the encroachment of dental caries?

Dr. Frank Sage opened the discussion of this theme. He thought that vitality had nothing to do with either the resistance to, or the prevention of decay, as in the dead body the teeth do not decay. He said that the formation of secondary dentine would lead us to suppose that there is some vital force.

Dr. Kempton remarked that the same conditions do not obtain in the skeleton as in the living mouth. When death occurs after a meal the gastric juice, which is acid, attacks the coats of the stomach. Hence he thought, that vitality of the tooth does resist decay.

Dr. Jay said that sometimes we see teeth that are badly neglected which nevertheless do not decay, while others properly cared for succumb to caries. In the latter case it is difficult to account for it, unless the person does not get enough bone-producing material in the food.

Dr. J. Taft said that the living teeth resist the attacks of decay producing agents better than dead ones. Soon after the loss of vitality in a tooth decomposition sets in. While living, the tissue retains its integrity, for vitality is the bond of union between the elements composing the tissue. Tooth structure is made up of both inorganic and organic substances, the former being held together by chemical force. The teeth of early life when devitalized soon change color, because the organized constituents are present in greater proportion than later in life. A dead tooth, solid, well made, in good surroundings, will better withstand decay than a living one, composed of a larger proportion of organic tissue and in bad surroundings. He cited in proof of this what Magitot calls "the zone of resistance" which is formed by filling up of the tubuli, and is a process demonstrating the operation of vitality.

Dr. Sage said that at first he thought the discussion was about vitality in general, and specially about the teeth.

Dr. W. H. Sillito said that the question at issue had not yet been touched. The resistance of vitality being admitted in the question.

Dr. A. O. Rawles asked; Can we measure the resistance of vitality? The susceptibility of tissue to change of any kind is in proportion to the resistance of the general vitality. Admitting vitality to be uniform, the surroundings of the teeth make a difference in the decay. The continuity of a tissue forms its principal resistance, though the maintainance of the circulation and affinities of tissues also enter into the the account. We cannot deal directly with such a subtle force as vitality, and can only argue from tangible things. The bond of union in dentine is destroyed in dental caries; vitality disappearing when nutrition ceases.

Dr. J. Taft said if we are to do away with subtle forces we must leave out of scientific discussion chemical force, specific gravity, &c.

Fourth Subject. Removal of the first permanent molars. When indicated?

Dr. Frank A. Hunter opened this discussion by advocating the extraction of these teeth where certain conditions exist. These are: before the twelfth year, when the pulp is dead, when the arch is crowded, and if the decay is too large to make a permanent filling. It is useless to fill these teeth under these circumstances, for the space they occupy is otherwise valuable.

Dr. J. Taft said that sometimes the removal is admissible, while again, no matter how badly they are decayed, their removal is a mistake. Nine times out of ten extraction is injurious. Removal of the first molars, if premature, interferes with mastication and proper occlusion of the teeth. The second molar will fall forward, and only one part of it antagonize with the opposing tooth. Their removal is indicated when diseased extensively and curative treatment is impossible. He objected to the use of the terms "six year molar," "dens sapientiæ," "wisdom tooth," &c.

Dr. A. Berry, of Cincinnati, agreed with Dr. Taft in the preservation of the first molars. It is well to retain them to keep the other teeth in position.

Dr. James Taylor also coincided with Dr. Taft that the extraction disturbed the whole antagonism. When the teeth are irregularly disposed, find the exact condition of the first molar. At the twelfth year the crown of the second molar is formed and the tooth is about to take its position. If the first one is extracted there is great probability of the second tipping forward and interfering with occlusion. As a rule he preferred extraction to filling when the teeth are crowded.

Dr. E. Osmond, of Cincinnati, coincided with Dr. Taylor. A father, large frame, and mother small, will have children whose teeth will be crowded. The nourishment of the teeth is not perfect when the arch is crowded. In considering such cases we must take into account the character of the jaw as well as the condition of the teeth.

Dr. G. W. Keely said he appreciated the value of the first molars. It often happens that children from seven to eight years old have their first molars extensively decayed and pulps exposed. Not more than one in a hundred such cases will be permanent when treated and filled. For the better illustration of his remarks Dr. Keely exhibited paintings and models. The first case he spoke of was a young girl, whose upper canines came through above and in front of the arch. The two superior first molars were extracted though they were sound, the first molars of the lower jaw badly decayed, were also removed, the four teeth being taken out at one sitting. The case is still in hand, with the expectation that the canines will come into their places without the use of force.

The second case exhibited the ill effects of the unnecessary removal of the first molar on the upper right side. All of the teeth on that side fell back, leaving considerable space between the central incisors. He said it was difficult to speak on the subject of the removal of the first molars without touching upon irregularity, because the two subjects interlace.

Dr. Dennis, asked if the canines appeared inside the arch should they be left to nature?

Dr. Keely replied that in such a case he would pull them forward. It is very rare to see them come through behind the arch, in the first case the use of an appliance is unnecessary. He advised young men, especially, not to take out the deciduous molars before the permanent ones had come through.

Dr. J. Taft said that in most cases the space occupied by the first molars, when extracted, is not completely filled by the remaining teeth coming together. The locality is unfavorable for mastication on account of pressure upon the gums; the consequence being that the teeth become the seat of deposits.

Dr. Osmond said that he preferred the extraction of a bicuspid because a molar left too large a space.

Dr. H. A. Smith thought that the first molars, as a general thing, were condemned by the profession.

Dr. J. Taft had observed throughout the discussion that a uniformity of opinion by the members was due to a principle which all dentists seem to recognize.

Seventh subject: To what extent should other materials than gold be used in filling teeth.

Discussion was opened by Dr. James Taylor. He thought that the profession had erred during the past ten or fifteen years in endeavoring to put permanent fillings of gold in the mouths of young persons, gold being too good a conductor of heat. There are other substances that could be used to serve a temporary purpose. He regarded tin as a filling material second only to gold.

Dr. Ulrey said that not more than fifty per cent. of teeth should be filled with gold. The dentine hardens better under a plastic material or tin, than it does if gold is used.

Dr. Taylor said that he had frequently met with many teeth devitalized by the use of gold, when oxide-chloride of zinc would have saved them.

Dr. J. C. McCampbell said that to all intents and purposes, a layer of oxy-chloride of zinc under the gold filling would answer the requisites of non-conduction.

Dr. Taylor said that during a prolonged attack of typhoid fever, the teeth are very likely to suffer more or less from the debilitated state of the system. When the surfaces of a cavity are in a condition of exalted sensibility, he thought it more proper to put in either tin or oxy-chloride of zinc. The profession some years ago introduced twenty fillings of tin to one put in now.

Dr. H. M. Reid, of Cincinnati, asked Dr. Taylor what proportion of teeth filled with tin were successful cases during his experience of forty years.

Dr. Taylor replied that as a usual thing tin is more adaptable than gold, and although it is susceptible of slight oxidation it preserves the teeth in many instances. The use of cohesive foil in an irregularly shaped cavity will not preserve the tooth with the same success obtained in the use of tin.

Dr. Jay had listened with much interest to the remarks upon this subject, and in his estimation it comprehends many points of vital interest and importance to all of us. He thought that gold is not the best material to use in all the cases that came under observation. If the teeth are strong, compact and healthy, gold in all probability is the best material to use. He said that he had not had a very extensive experience in the use of tin foil. When the structure of the teeth is chalky, and they are attacked by the white decay, he doubted very much whether there was any material that would save them for any length of time, and in such cases tin is no doubt the proper substance to use. For children between the ages of ten and fourteen years it is better to employ tin, because if filled with gold it will at no distant period require to be taken out and the cavities refilled. The malleting of gold into young teeth which are sensitive, is very apt to produce an inflammation of the periosteum, and besides, the gold by conducting thermal changes will serve to keep up a constant irritation. He said he would not use gold any more for filling children's teeth, and instead would use gutta percha, it being preferable to oxy-chloride of zinc, because the latter is washed away by the fluids of the mouth.

Dr. Rawls said that this subject has been occupying the attention of the profession for some four or five years past, the profession in the East more especially giving it their attention. As he understood it, it is the ability of the operator to correctly diagnose the case under his immediate observation, then the treatment to be pursued will naturally suggest itself. It is not only necessary to thoroughly comprehend the surrounding circumstances, and in addition have a knowledge of the constitutional condition of the patient. In the teeth of young persons, the structure is soft, in which case the use of a plastic material gives the teeth an opportunity to reach their normal density. In these teeth we find that the tubuli are large, the tendency being as age progresses to become filled up and consolidated. He thought that the proper course to pursue in such cases was to use some temporary material. When this was done he considered the operation as much finished as though it had been performed with gold in the usual manner, and thought that it was wrong to put in gold where it was contraindicted, merely for the sake of getting more money for the work.

Dr. J. Taft said that the material should be selected to meet the demands of the case, and thought that in some children's teeth gold could be used. He thought that it did not exert a good influence upon the young members of the profession to argue against large and tedious operations.

Fifth subject: "Pathology and treatment of exposed pulps."

Discussion opened by Dr. J. Taft. In the diagnoses of the case, it is necessary to consider systemic conditions. Sometimes malarial poisoning of the blood renders it impossible almost to treat with success exposed pulps. The local condition is also necessary in the diagnosis, whether the fluids of the mouth are vitiated or not, extent of exposure, degree of inflammation, whether there is pus or not, and its quantity and character. The healthy pulp is of a pink color, if actively inflamed it is red and swollen, indicating depletion or counter irritation as the treatment. If there is a high degree of inflammation, the pulp is of a brown color.

Pepsin paste applied to the pulp will digest the diseased portion in twenty-four or thirty-six hours, the pulp reassuming its healthy color.

Dr. Jay asked Dr. Taft what is the method of preparing the pepsin paste.

Dr. Taft replied that the liquid and powered pepsin could be mixed into a paste, or the powder mixed with dilute muriatic acid.

Dr. Wm. Van Antwerp remarked that an extract made from the rind of the ordinary paw-paw will digest a beef-steak, and could with great propriety be used instead of pepsin to digest the effete portion of a diseased pulp. During its action, the extract does not have any effect upon the living tissue that remains. As soon as a pulp regains its normal color and gets back into its place, flow over it something that is non-irritant, and will be acceptible to it, all space over the pulp being completely occupied by the capping. The oxy-phosphate of zinc is a non-irritant material, and can be used with succ-ss in these cases.

Dr. Fletcher noted several cases he had met with, of hypertrophied pulps, in one of which there was so little sensibility that the patient could without pain masticate soft substances upon it.

Dr. Van Antwerp did not think that Dr. Fletcher's case was a pulp, but rather a fungus growth of the gum coming up between the teeth and filling the cavity of decay.

Dr. J. Taft said that when the pulp was so hypertrophied its degeneration made it hardly worth the trouble to attempt to save it, the nerve tissues in it being almost totally destroyed; extirpation is the treatment.

Dr. E. Osmond said that these growths are largely supplied with blood vessels, and if covered up are very apt to produce trouble. It is not necessary to devitalize them, for they can be cut away with little or no pain.

Dr. J. Taft said that sometimes a calcareous deposit in the tooth pulp will occupy the pulp chamber, often causing neuralgia and prevent the success of conservative treatment.

Dr. Van Antwerp said that people living in lime stone regions usually possess a more perfectly developed boney structure, their teeth also being of a better quality. This is probably due to their using water impregnated with lime.

